# Adult renal function is modified by perinatal taurine status in conscious male rats

**DOI:** 10.1186/1423-0127-17-S1-S31

**Published:** 2010-08-24

**Authors:** Sanya Roysommuti, Pisamai Malila, Dusit Jirakulsomchok, J Michael Wyss

**Affiliations:** 1 Department of Physiology, Faculty of Medicine, Khon Kaen University, Khon Kaen 40002, Thailand; 2 Department of Cell Biology, University of Alabama at Birmingham, Birmingham, AL 35294, USA

## Abstract

Perinatal taurine exposure influences renal function in adult female offspring. This study tests the hypothesis that prenatal rather than postnatal taurine exposure alters renal function in adult conscious male rats. Female Sprague Dawley rats were fed normal rat chow and tap water alone (Control), tap water containing 3% β-alanine (taurine depletion, TD) or tap water containing 3% taurine (taurine supplementation, TS) either from conception until delivery (fetal period; TDF or TSF) or from delivery until weaning (lactation period; TDL or TSL). After weaning, male offspring were fed with the normal rat chow and tap water *ad libitum*. At 7-8 weeks of age, renal function was studied in conscious, restrained rats. Mean arterial pressures were slightly higher in rats receiving taurine supplementation during either the fetal or lactation periods (compared to Control and TD groups), but heart rates were not significantly different among groups. Effective renal blood flows were lower in TDF, TDL, and TSF rats (TDF 4.6±0.8 ml/min/g kidney weight (KW), TDL 3.0±0.9 ml/min/g KW, and TSF 2.8±0.7 ml/min/g KW) than in TSL (7.7±0.9 ml/min/g KW) or Control rats (7.3±1.6 ml/min/g KW). These differences were correlated with significant increases in renal vascular resistance in TDF, TDL, and TSF groups compared to TSL and Control rats. In contrast, glomerular filtration rates were not significantly different among groups. Although basal water and sodium excretion were slightly lower in TDL and TSF rats compared to other groups, their diuretic and natriuretic responses to an acute saline load were not different from Control. The present data indicate that in adult male rats, both perinatal supplementation and depletion of taurine can alter renal hemodynamics, and these effects are differentially time-dependent.

## Background

Taurine, 2-aminoethanesulfonic acid, is present at a high concentration in many organs including liver, brain, heart, kidneys, and reproductive organs [1].  In animals and humans, taurine contributes importantly to cell growth and development in early life and cytoprotection and cell volume regulation throughout life [2].  Taurine content in various tissues is highest during the perinatal period, and it modestly declines with advancing age.  Taurine is an essential β-amino acid in fetal development due to limitation of endogenous biosynthesis [3].  Thus, maternal availability of taurine through the placenta is the only supply of taurine in early life.

Perinatal taurine depletion induces multiple organ damage in neonates and adult rats.  While the mechanisms underlying these adverse effects remain ambiguous, a decrease in β-amino acid taurine content likely leads to the adult disorder [3-6].  Renal dysfunction related to age, diabetes mellitus, hypertension and obesity are inversely correlated to body taurine content [7].  Thus, taurine supplementation could prevent age-related renal damage, sugar-induced hypertension, ethanol-induced hypertension, and drug-induced diabetes [2,3,8-10].   A decrease in taurine content can be partly replenished by taurine supplementation or diets high in taurine like fish [7,11].

Perinatal taurine depletion impairs renal function in adult female rats.  In contrast, perinatal taurine supplementation appears to increase renal sodium excretion as tested by an acute saline load in these female offspring [12].  Tubular sodium reabsorption also decreases in these animals.  Moreover, perinatal taurine depletion heightens sympathetic nerve overactivity [13] and renal dysregulation [12] induced by a high sugar intake in adult rats.

In the rats, nephrogenesis is complete before birth and thus the nephron number cannot be increased later [14,15].  However, differentiation and maturation of renal function continue several weeks after delivery.  It seems likely that the programming of adult renal function may differ between prenatal and postnatal life.  This study tests the hypothesis that prenatal rather than postnatal taurine exposure alters renal function in adult conscious male rats.

## Materials and methods

Sprague Dawley (SD) rats were bred from the animal unit of Faculty of Medicine, Khon Kaen University and maintained at constant humidity (60 ± 5%), temperature (24 ± 1°C), and light cycle (0600-1800 h).  Female SD dams were fed normal rat chow and drank tap water (Control), water containing 3% alanine (taurine depletion, TD) or water containing 3% taurine (taurine supplementation, TS) either from conception until delivery (fetal or prenatal period, TDF or TSF) or from delivery until weaning (lactation or postnatal period, TDL or TSL).  After weaning, the male offspring were fed with the normal rat chow and water *ad libitum*.  All experimental procedures were preapproved by the Khon Kaen University Animal Care and Use Committee and were conducted in accordance with the National Institutes of Health guidelines.

At 7-8 weeks of age, under sodium pentobarbital anesthesia (Nembutal 50 mg/kg, i.p.), all male rats were implanted with femoral arterial, venous, and bladder catheters.  Forty-eight hours later, arterial pressure was continuously recorded in conscious, restrained rats,  a condition to which all rats had been acclimated for one week (3 hours per day) prior to the experiment.  Renal function was studied before, during, and after an intravenous isotonic saline infusion (a mixture of 0.5% inulin and 0.5% PAH in isotonic saline, 5% of body weight, 0.5 ml/min).  At the end of experiments, all animals were sacrificed and kidney (KW) and heart (HW) weights were measured.

Urine volumes were measured gravitationally, urine and plasma sodium by flame photometry and urine and plasma inulin and p-aminohippuric acid (PAH) by colorimetry.  Glomerular filtration rate (GFR) was estimated by inulin clearance, effective renal blood flow (ERBF) by PAH clearance and hematocrit, renal vascular resistance (RVR) by MAP/ERBF, fractional water excretion by a urine flow to GFR ratio (FE_H2O_), and fractional sodium excretion by the ratio of urine sodium excretion to filtered sodium load (FE_Na_).

All data were expressed as mean ± SEM and were statistically analyzed using one-way ANOVA and appropriate post hoc tests (Duncan’s Multi-Range) with a significant criterion of p < 0.05.

## Results

**Figure 1:**
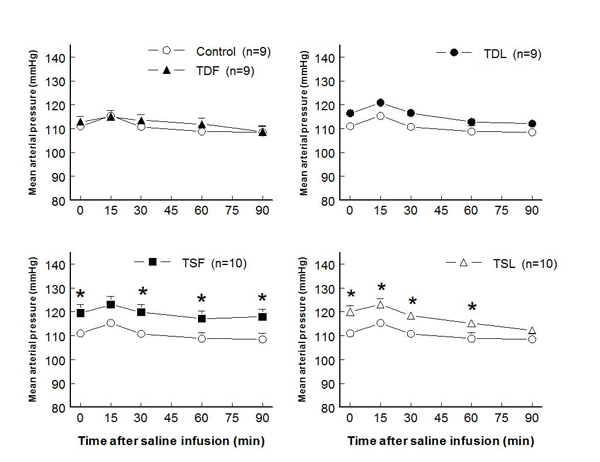
**Mean arterial pressure before and after acute saline infusion in adult conscious rats** (* P < 0.05 when compared to control of same time; see text for abbreviations)

**Figure 2:**
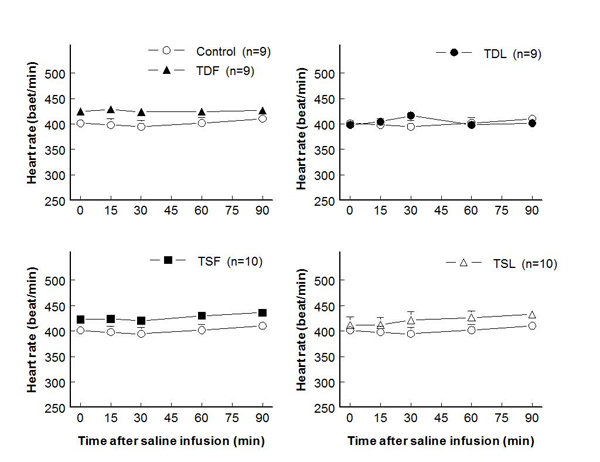
**Heart rate before and after acute saline infusion in adult conscious rats** (no significant difference when compared to control of same time; see text for abbreviations)

Prenatal or postnatal taurine under or over exposure did not alter body or kidney weights in all mature rats.  While heart weight and heart to body weight ratio significantly increased in TDF, these measures were not significantly different among the other groups (Table 1).  Either prenatal or postnatal taurine supplementation slightly and significantly increased mean arterial pressures at rest and after a saline load when compared to control rats (Fig. 1).  In contrast, prenatal or postnatal taurine depletion did not alter the mean arterial pressures throughout the study.  Heart rates were not significantly different among groups (Fig. 2).

**Table 1:** Adult body (BW), kidney (KW), and heart weights (HW) in experimental groups.

Treatment	BW(g)	KW(g)	HW(g)	KW/BW(%)	HW/BW(%)
Control(n=9)	212 ± 4	1.85 ± 0.09	0.71 ± 0.03	0.87 ± 0.04	0.33 ± 0.01
TDF (n=9)	223 ± 7	1.95 ± 0.10	0.89 ± 0.00*	0.87 ± 0.03	0.40 ± 0.01*
TDL (n=9)	214 ± 6	2.00 ± 0.10	0.73 ± 0.01	0.93 ± 0.04	0.34 ± 0.01
TSF (n=10)	216 ± 3	2.02 ± 0.09	0.72 ± 0.03	0.93 ± 0.03	0.34 ± 0.01
TSL (n=10)	215 ± 6	1.83 ± 0.09	0.74 ± 0.03	0.85 ± 0.04	0.35 ± 0.01

Data are Mean ± SEM. * P < 0.05 vs. all other groups. See text for abbreviations.

**Figure 3:**
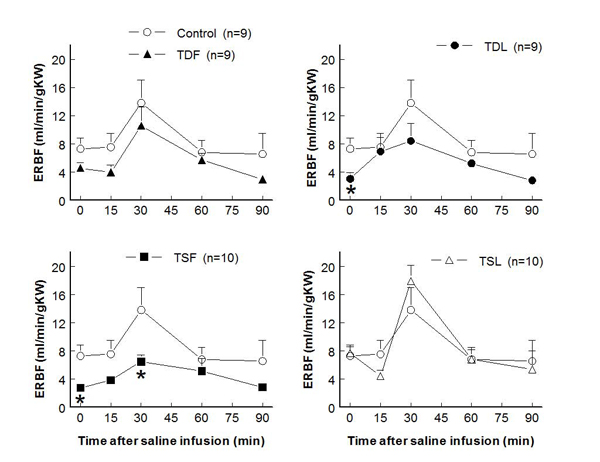
**Renal blood flow before and after acute saline infusion in adult conscious rats** (* P < 0.05 when compared to control of same time; see text for abbreviations)

**Figure 4:**
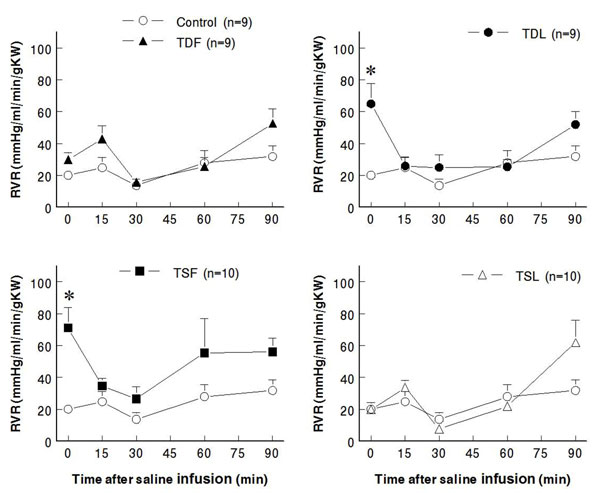
**Renal vascular resistance before and after acute saline infusion in adult conscious rats** (* P < 0.05 when compared to control of same time; see text for abbreviations)

**Figure 5:**
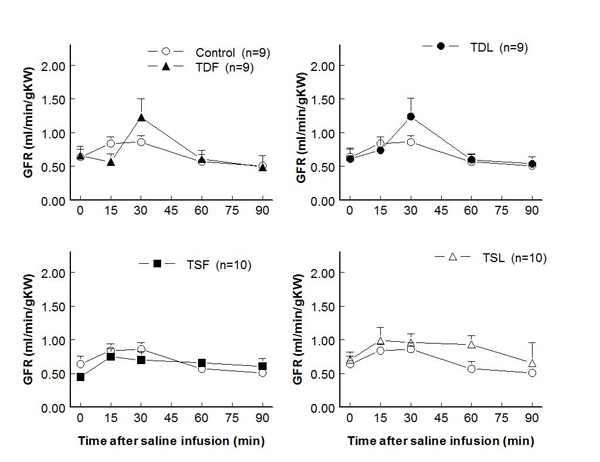
**Glomerular filtration rate before and after acute saline infusion in adult conscious rats** (no significant difference when compared to control of same time; see text for abbreviations)

**Figure 6:**
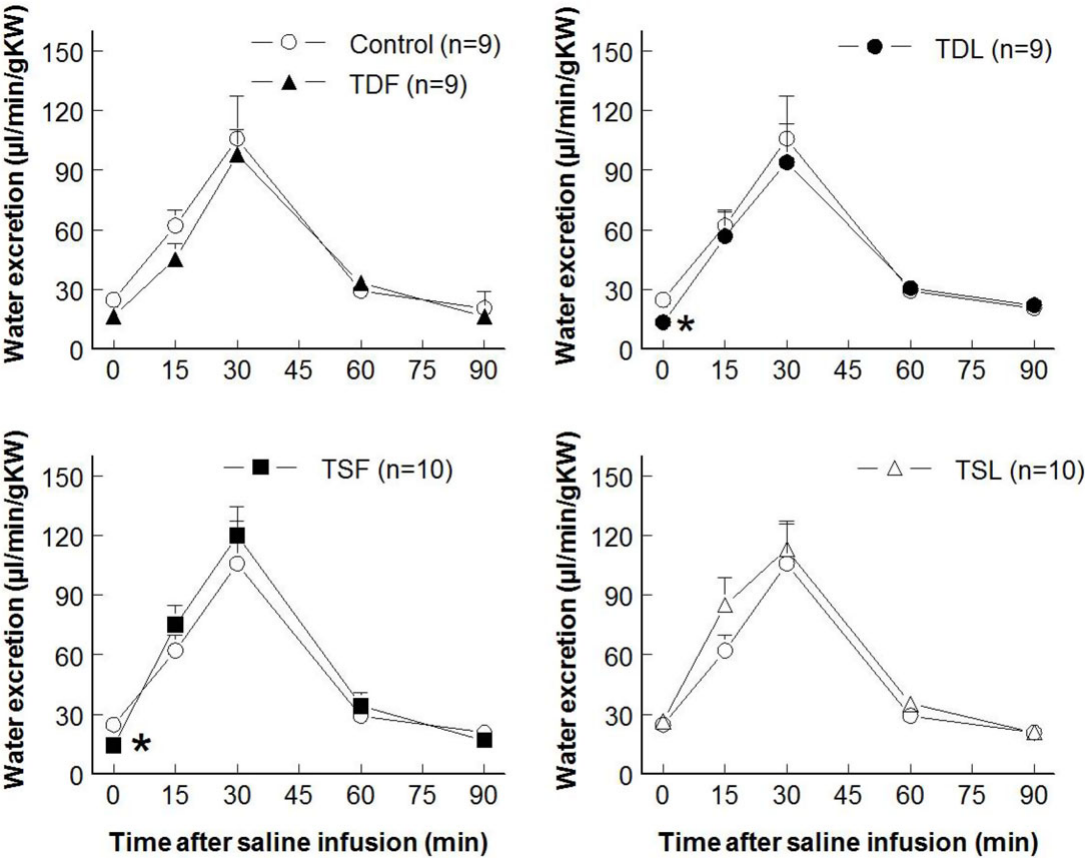
**Renal water excretion before and after acute saline infusion in adult conscious rats** (* P < 0.05 when compared to control of same time; see text for abbreviations)

**Figure 7:**
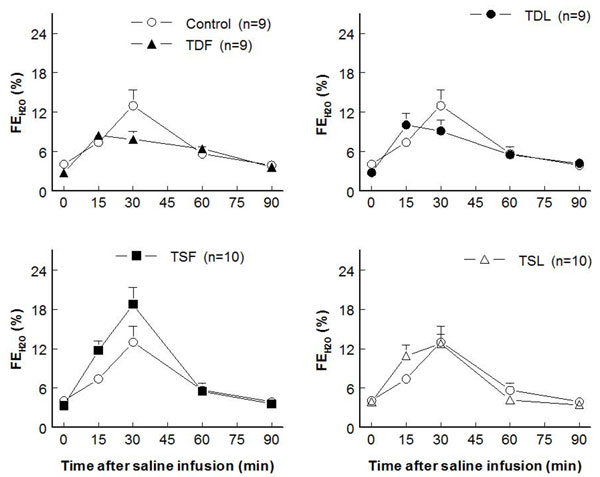
**Fractional water excretion (FE_H2O_) before and after acute saline infusion in adult conscious rats** (no significant difference when compared to control of same time; see text for abbreviations)

**Figure 8:**
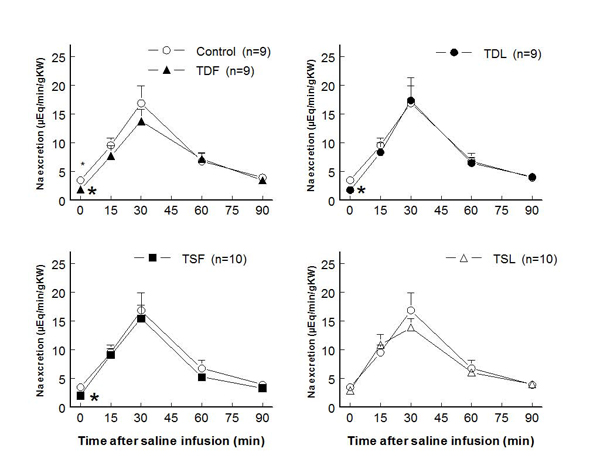
**Renal sodium excretion before and after acute saline infusion in adult conscious rats** (* P < 0.05 when compared to control of same time; see text for abbreviations)

**Figure 9:**
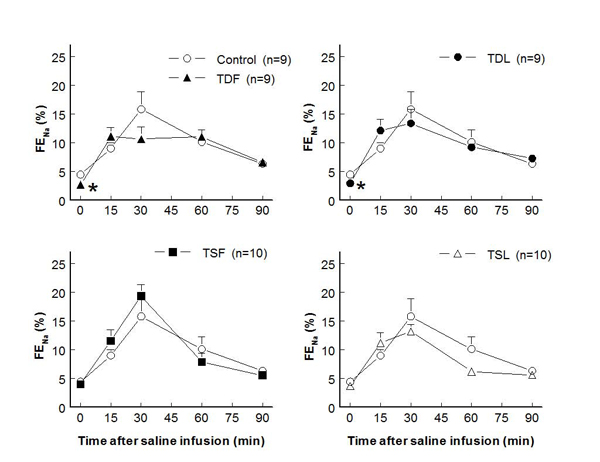
**Fractional sodium excretion (FE_Na_) before and after acute saline infusion in adult conscious rats** (* P < 0.05 when compared to control of same time; see text for abbreviations)

Compared to control (7.3±1.6 ml/min/g KW), effective renal blood flows significantly decreased in TDL and TSF but not  in TDF and TSL rats (at rest: TDF 4.6±0.8 ml/min/g KW, TDL 3.0±0.9 ml/min/g KW, TSF 2.8±0.7 ml/min/g KW, TSL 7.7±0.9 ml/min/g KW; p < 0.05) (Fig. 3).  These changes were inversely correlated with significant increases in renal vascular resistance in TDL and TSF, as well as a trend to increase in TDF groups (Fig. 4).  Glomerular filtration rates were not significantly different among groups both at rest and after an acute saline load (Fig. 5).  Water excretion at rest was slightly but significantly decreased in TDL and TSF, while sodium excretion was significantly decreased in TDF, TDL, and TSF when compared to control.  However, water excretion, fractional water excretion and GFR responses to the acute saline load were not significantly different among groups (Fig. 6, 7, 8).  Fractional sodium excretion significantly decreased at rest in perinatal taurine-depleted but not in perinatal taurine-supplemented rats (Fig. 9), but irrespective of baseline difference, the groups displayed similar FE_Na_ responses to an acute saline load.

## Discussion

Previous experiments demonstrate that over or under exposure to taurine from conception until weaning (perinatal life) alters long-term regulation of renal function and arterial pressure [12].  The present study demonstrates that both prenatal and postnatal taurine manipulation can impair renal function in adult male rats.  Prenatal taurine supplementation and postnatal taurine deficiency had many similar adverse effects on the adult renal function, including decreased renal blood flow and resting water and sodium excretion, and increased renal vascular resistance and resting tubular sodium reabsorption.  However, both prenatal and postnatal taurine supplementation markedly blunted renal pressure-diuresis/natriuresis in the adult.

Prenatal taurine deficiency produces low birth weight in animals, especially cats.  These animals also display many abnormalities including organ damage [2,3,12]. The only difference in organ weights in the current study was the slight increase in heart weight in TDF.  These data indicate that the taurine manipulations shortly during prenatal or postnatal period do not dramatically alter growth of the animal or compromise overall organ growth.  Further, heart rates were not different among groups, indicating that the taurine manipulations do not have a gross effect on this parameter; however, whether cardiac contractility and/or stroke volume differ among groups should be directly tested in the future.

In rats, nephrogenesis is completed before birth, but renal differentiation and maturation continue postnatally [14,15].  In the present study, postnatal taurine deficiency impairs renal function especially renal hemodynamics and resting water and sodium excretion slightly greater than prenatal deficiency.  This suggests that taurine exposure has a significant role in the development of renal function in early postnatal life.  In the present study, all rats were supplied by a normal rat food from weaning to the end of experiment, thus, the changes in the pre and postnatal taurine deficient rats was not fully corrected by post-weaning normalization of taurine in the diet.  In the present study, prenatal (compared to postnatal) taurine-depleted rats displayed relatively minor deficits in renal blood flow and sodium excretion, suggesting that postnatal taurine exposure may partially restore renal function in these rats.

Our previous study demonstrates that perinatal taurine deficiency depresses autonomic nervous system function in adult male rats and that this can be reversed by high sugar intake post-weaning [13].  This suggests that an early life dietary intervention can reverse renal deficits attributable to taurine deficiency.

Our previous data demonstrates that perinatal taurine depletion from conception until weaning does not alter arterial pressure or heart rate in adult male and increases arterial pressure but not heart rate in female rats.  In addition, high sugar intake induces hypertension only in male rats [16].  The present data extend these findings, demonstrating that selective prenatal or postnatal taurine deficiency has no long-term effect on arterial pressure and heart rate at this stage of adult life.  However, taurine contributes importantly to nervous system growth and differentiation [3,17,18], and abnormal autonomic nervous system function underlines sugar-induced hypertension in perinatal taurine-depleted male rats [13].  In addition, high dietary sugar be itself does not increase arterial pressure in most studies.  Thus, altered, early-life taurine exposure may be a co-factor for other hypertensive agents and thereby contribute to hypertension. Sympathetic nervous system overactivtiy also directly impacts target organs and/or indirectly affects them via other mechanisms, e.g., the renin-angiotensin system.

Long-term arterial pressure control is mainly dependent on renal pressure-diuretic/natriuretic mechanisms [19-21].  In general, the kidneys will excrete sufficient water and sodium to reduce arterial pressure to normal levels.  In the present study, water and sodium excretion in responses to an acute saline load were not significantly different among groups despite higher arterial pressures in perinatal taurine-supplemented groups, suggesting that blunted renal pressure-diuretic/natriuretic mechanisms are present in this group as well as the taurine-depleted groups.

Perinatal malnutrition and low birth weight can lead to adult hypertension, renal dysregulation, obesity, diabetes mellitus and other abnormalities [22].  These early life abnormalities appear to cause cell adaptation that prefers anabolism over catabolism in adults.  Without a sufficient diet and activity control, such individuals are at higher risk of obesity, hypertension, and other cardiovascular diseases.  Although obesity and insulin resistance [16] were not observed in the present animals, renal dysregulation was confirmed.  Further, the present data suggest that if these animals were studied when aged (e.g., 12 months or beyond), early deficits in renal, glucose/insulin and autonomic dysregulation may lead to insulin resistance, diabetes, and hypertension.

During pregnancy, mothers need more nutrients for their own lives and their fetuses.  Many commercial dietary supplements contain sufficient taurine for these individuals [9,23,24], and taurine plays many physiological roles throughout life.  Our previous studies indicate that taurine supplementation from conception until weaning significantly increases arterial pressure and renal vascular resistance and decreases renal blood flow in adult female rats [12].  The present study further demonstrates that both prenatal and postnatal taurine over exposure contributes to similar effects in male rats.  Prenatal over nutrition has also been reported to induce obesity and hypertension in the elderly [25-27], and the current data indicate that excess taurine may have some adverse side effects.

## Conclusion

Previous experiments indicate that perinatal taurine over or under exposure influences arterial pressure and renal function in mature male and female rats.  The present results extend these findings and demonstrate that timing of dietary taurine exposure during the perinatal period can adversely shape adult renal function and arterial pressure.  Thus, taurine exposure appears to be an important consideration for pregnant women and neonates.

## List of abbreviations used

CW: control with water intake alone; CG: control with high sugar intake; TD: taurine depletion; TDF: prenatal taurine depletion; TDL: postnatal taurine depletion; TS: taurine supplementation; TSF: prenatal taurine supplementation; TSL: postnatal taurine supplementation; BW: body weight; HW: heart weight; KW: kidney weight; SD: Sprague Dawley; i.p.: intraperitoneal; SEM: standard error of means; Na: sodium; PAH: p-aminohippuric acid; GFR: glomerular filtration rate; ERBF: effective renal blood flow; RVR: renal vascular resistance; MAP: mean arterial pressure; FE_H2O_: fractional water excretion; FE_Na_: fractional sodium excretion

## Competing interests

The authors declare that they have no competing interests.

## Authors’ contributions

Sanya Roysommuti: research proposal design, data analysis, article preparation, correspondence. Pisamai Malila: research proposal preparation, data collection and analysis. Dusit Jirakulsomchok: research consult, article preparation. J Michael Wyss: research consult, article preparation.
